# Prion protein genotype survey confirms low frequency of scrapie-resistant K222 allele in British goat herds

**DOI:** 10.1136/vr.103521

**Published:** 2016-01-11

**Authors:** W. Goldmann, E. Marier, P. Stewart, T. Konold, S. Street, J. Langeveld, O. Windl, A. Ortiz-Pelaez

**Affiliations:** 1 The Roslin Institute and R(D)SVS University of Edinburgh, Easter Bush, Midlothian EH25 9RG, UK; 2 Animal and Plant Health Agency Weybridge, Woodham Lane, Addlestone, Surrey KT15 3NB, UK; 3 Central Veterinary Institute part of Wageningen UR (CVI) Department of Infection Biology, P.O. Box 65, 8200 AB Lelystad, The Netherlands

**Keywords:** Scrapie, Prion protein, Goats, Gene polymorphisms, Resistance, Brain diseases

## Abstract

Scrapie in goats is a transmissible, fatal prion disease, which is endemic in the British goat population. The recent success in defining caprine *PRNP* gene variants that provide resistance to experimental and natural classical scrapie has prompted the authors to conduct a survey of *PRNP* genotypes in 10 goat breeds and 52 herds to find goats with the resistant K222 allele. They report here the frequencies in 1236 tested animals of the resistance-associated K222 and several other alleles by breed and herd. Eight animals were found to be heterozygous QK222 goats (0.64 per cent genotype frequency, 95 per cent CI 0.28 to 1.27 per cent) but no homozygous KK222 goats were detected. The K222 allele was found in Saanen, Toggenburg and Anglo-Nubian goats. The fact that only a few goats with the K222 allele have been identified does not preclude the possibility to design and implement successful breeding programmes at national level.

Scrapie in goats and sheep is a transmissible, fatal disease with an average onset age of two to five years. It belongs to the group of transmissible spongiform encephalopathies (TSEs), also known as prion diseases, which include bovine spongiform encephalopathy (BSE) in cattle, chronic wasting disease in cervids and Creutzfeldt-Jakob disease in humans (Chesebro 2003). Scrapie can have incubation periods of a few years, during which the animals appear clinically normal and can transmit the infectious agent to herd-mates. Scrapie appears in two major disease forms, termed classical and atypical, and is distinguished by, among others, epidemiology, clinical disease presentation, brain pathology and genetic association of susceptibility.

Prion disease susceptibility is controlled by host genetics, primarily by the prion gene *PRNP*. The protein encoded by this gene, PrP or prion protein, exists in many variant forms in goats and sheep, most of them differ only by a single amino acid change in their protein sequence. In sheep, these PrP variants have been used in breeding programmes such as the National Scrapie Plan in Great Britain. Ovine PrP variants are generally defined by their amino acids in codons 136, 154 and 171, for example, ARQ. The various resulting genotypes have different association with classical scrapie susceptibility or resistance. Consequently, several countries set up breeding programmes in which animals with highly susceptible genotypes (VRQ/VRQ and VRQ/ARQ) were eliminated and animals with highly resistant genotypes (ARR/ARR and ARQ/ARR) (for review, see Goldmann 2008) were propagated. Recent data for the prevalence of scrapie have shown that the number of classical scrapie cases in sheep is consistently falling in Great Britain and other EU Member States (EFSA BIOHAZ Panel 2014).

In goats, *PRNP* alleles also exist, but the encoded PrP variants are defined by amino acid changes in codon positions different from sheep (for review, see Vaccari and others 2009). With regard to the sheep *PRNP* allele terminology, goats are normally ARQ or AHQ. Worldwide, there are well over 30 caprine PrP variants known, but many are relatively rare and their association with scrapie is unspecified. Common PrP variants in UK goat breeds are defined by polymorphisms I142M, R154H, R211Q and Q222K, which abbreviated to IRRQ (I_142_R_154_R_211_Q_222_) defines the caprine wild-type PrP. Other polymorphisms that are regularly found in British breeds are G127S, N146S and P240S, the last one being positioned outside the sequence of the mature PrP protein. Goats and sheep share at least 10 amino acid polymorphisms, of which R154H is the most noticeable as it is associated with susceptibility to atypical scrapie in both species (Moum and others 2005, Arsac and others 2007, Colussi and others 2008).

Protective effects of varying degree to experimental and natural classical scrapie exposure are seen in goats with genotypes carrying the S127, M142, S146, D146, H154, Q211 or K222 alleles. Experimental scrapie challenge of QK222 and KK222 genotypes using the most effective intracerebral route leads to disease in only a small number of animals after very prolonged incubation periods compared with the wild-type QQ222 genotype (Lacroux and others 2014). However, no disease was transmitted after oral exposure to scrapie or BSE (Corbière and others 2013, Lacroux and others 2014, Aguilar-Calvo and others 2015). The protective effect of K222 is supported by epidemiological evidence confirming the scarcity of K222 carriers in natural scrapie cases (Acutis and others 2006, 2012, Vaccari and others 2006, Barillet and others 2009), although there may be exceptions to this association (Fragkiadaki and others 2011, Kanata and others 2014). The K222 allele is present in the most important dairy breeds (e.g. Saanen) although at rather low frequencies in most European countries, and it was particularly low in a survey of British goats conducted between 2007 and 2009 (Goldmann and others 2011). Strong evidence from case-control studies of Cypriot goat herds support the protection against classical scrapie conferred by the S146 and D146 alleles. (Papasavva-Stylianou and others 2007, 2011, Ortiz-Pelaez and others 2014). In the UK, only S146 has been found, and in larger numbers only in Boer goats (Goldmann and others 2011). White and others (2012) have reported that after oral scrapie challenge of NS146 heterozygotes, all animals survived without developing disease for 40 per cent longer (at which point the experiment was terminated) than wild-type homozygotes, which had a 100 per cent attack rate, suggesting that the protective effect of this amino acid change may not be restricted to Cypriot scrapie strains. Moreover, the amino acid variation asparagine or serine in codon 146 has been found in sheep, goats and cattle. Association of amino acid changes with scrapie disease susceptibility in one species may also apply to another species, which makes the codon 146 polymorphism particularly interesting.

The goat population in Great Britain is small compared with other EU countries and contributes a small but growing fraction to the total livestock production. The commercial goat sector is specialised with most of the large holdings producing milk for on-farm processing or collection mainly for pasteurisation and cheese production. A few pedigree herds maintain the supply of purebred males to commercial herds that have developed and maintained their own genetic lines of crossbred females, selected according to their production traits. The majority of goats in Great Britain are family pets with one or two individuals kept for purposes other than farming (Ortiz-Pelaez and others 2012). Scrapie became a notifiable disease in 1993 in the UK in accordance with EU Council Directive 91/68/EC. Passive surveillance systems, that is, reporting of clinical suspect animals, led to the histopathological confirmation of 28 UK goat scrapie cases between 1976 and 2002 (Dustan and others 2008), while in the 12-year period from 2002 to 2013 with additional active surveillance, a total of 204 cases were pathologically confirmed, all belonging to the classical disease form (Ortiz-Pelaez and Arnold 2014). This number of British scrapie cases supports the consideration of selection programmes based on *PRNP* genetics similar to sheep.

The purpose of this study was to ascertain by means of a survey the current frequency of the resistance-associated K222 allele in British goats. This survey was also used to record other *PRNP* polymorphisms, which allowed a comparison of *PRNP* allele and genotype frequencies in the British goat population to a similar survey conducted by the authors five years ago.

## Materials and methods

### Herd selection and sample collection

The initial selection was based on ≥10 goats per holding using the list of herds in the Sheep and Goat Inventory 2012 in Great Britain, applying a multistage random sampling strategy. Within the herds, all males were sampled and additional females were chosen by the goat keeper to reach the maximum number of animals as determined by the sample size calculation (see below). Animals older than six months and intended to be kept as breeding animals were eligible for the survey.

The calculation for the number of herds to sample was based on the assumption that 10 per cent of the herds contained at least one animal with the 222K allele, a confidence level of 95 per cent confidence and 5 per cent desired accuracy of the expected prevalence. To calculate the sample size in each herd, a 2 per cent prevalence of the 222K in the whole population of goats was assumed with a 95 per cent confidence level and 5 per cent desired accuracy. Assumptions on animal and herd prevalence of the 222K allele in the British goat population were based on published (Goldmann and others 2011) and unpublished data (W. Goldmann, unpublished observations). With these assumptions, a maximum of 30 animals per herd (including all males) and 117 herds were required for this survey. With the current distribution of herd size in the sampling frame as per holdings with >10 goats listed in the Sheep and Goat Inventory 2012 (Defra UK website), approximately 2300 samples (one sample per animal) were required.

The survey was presented at the Goat Veterinary Society Annual Meeting in November 2012 (Ortiz-Pelaez and others 2013) and two calls for expressions of interest were published in the *British Goat Society Journal* (Clayton 2013, Ortiz-Pelaez 2013) in an attempt to increase participation. Participation in the survey was voluntary. In return, recruited goat keepers were offered free sampling, genotyping and a report with genotype results and animal details. Participants were advised to retain animals with resistant genotypes with the view to promote breeding for scrapie resistance in their herd. The selection of the farmers was adapted due to the difficulty in recruiting randomly selected farms. Farmers were first recruited from those who expressed an interest and met the minimum herd size. Subsequently, goat keepers were selected at random from the database of holdings (inventory 2012) and along with goat breeder associations and veterinarians contacted directly with the view to increase the returns, which resulted in recruitment of three farms with <10 goats. During the sampling visit, owner's consent was obtained via a signed acceptance of the sampling procedure in the conditions described earlier. They were also asked to fill in a questionnaire to obtain basic epidemiological data of the herd: production type and number of homebred and purchased goats by age, sex and breed.

Initially, a blood sample was collected from the jugular vein (max. 10 ml, in EDTA) for DNA extraction. It was later replaced by nasal swabbing whereby a cotton bud-sized sponge was gently rubbed along the nasal mucosa and placed in a liquid-containing tube to stabilise the DNA (Performagene Nasal PG-100 collection kit, DNA Genotek, Ontario, Canada). After a trial on several farms, the nasal swabbing was found to cause no harm and compared with insertion of a hypodermic needle was considered a refinement that did not constitute a regulated procedure under the Animal (Scientific Procedures) Act. It could, therefore, be carried out by farmers. From that moment, participating goat keepers received a kit with all the necessary equipment and paperwork to sample the animals and collect ear tag number/s, age, breed and sex. Goat keepers posted back the samples and paperwork to the project office.

### Genotyping

DNA was extracted from blood using the Qiagen DNeasy blood & tissue kit (Qiagen, Crawley, UK) or from the Performagene nasal swap (DNA Genotek, Ontario, Canada) as recommended by the manufacturers. PCR amplification of PrP coding region and sequencing were performed as described in Goldmann and others (2011). Two alternative PCR reactions were performed dependent on the quality of recovered DNA, one resulting in the full open reading frame (ORF) sequence and one producing codons 194 to 256 only to allow genotyping of codons 211 and 222.

## Results

**FIG 1:**
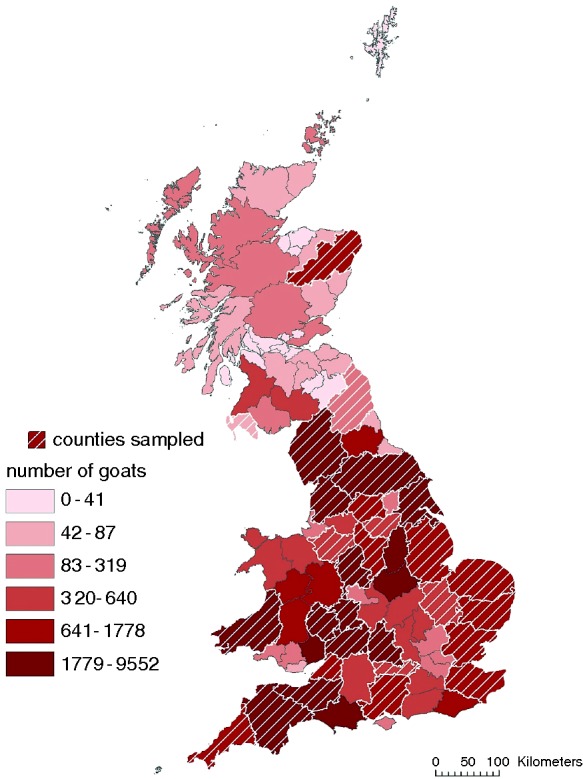
Distribution of participating goat keepers in Great Britain in relation to overall goat population, 2013

A total of 52 goat farms were included in the survey conducted between August 2013 and December 2014, which were located in England (49), Scotland (2) and Wales (1). Figure 1 shows the distribution of goat herds across Great Britain that were sampled in relation to the overall distribution of goat herds based on the 2013 goat inventory. Herd sizes ranged from 8 to 6651 goats (mean herd size 513; median 54). The goats were kept as breeding (12 herds, 22.6 per cent), production (38 herds, 71.6 per cent) or companion animals (3 herds, 5.7 per cent) encompassing 10 breeds (Saanen, Toggenburg, Alpine, Anglo-Nubian, Boer, Cashmere, Angora, pygmy, Golden-Guernsey and Bagot) and crossbreeds (see Tables 1 and 2). The production animal herds were split by breed type into 24 dairy (63.2 per cent), 8 meat (21 per cent) and 6 fibre (15.8 per cent) herds.

**TABLE 1:** Frequency of QK222 *PRNP* genotypes of goats by production type

	Breeding	Production			Pet	Other	Total
		Dairy	Meat	Fibre			
n (%)	255 (20.6)	573 (46.4)	170 (13.8)	171 (13.8)	42 (3.4)	25 (2)	1236 (100)
QK222 (%)	3 (1.1)	5 (0.8)	0	0	0	0	8 (0.6)
QQ222	252	568	170	171	42	25	1228

**TABLE 2:** Frequencies of QK222 *PRNP* genotypes in goat breeds and other *PRNP* gene variation by breed

Breed	Alpine	Anglo- Nubian	Angora	Boer	Cashmere	Golden-Guernsey	Pygmy	Saanen	Toggenburg	SaanenX, ToggenburgX	Others*	All
Herds n	11	15	5	14	1	4	2	24	17	10	5	52
Goats n	51	120	140	254	29	40	29	211	183	140	39	1236
QK222 n (%)	0	2 (1.7)	0	0	0	0	0	4 (1.9)	1 (0.5)	1 (0.7)	0	8 (0.6)
*Other detected polymorphisms*												
								R101				R101
						G102						G102
	S127			S127				S127	S127	S127		S127
			M142	M142			M142	M142	M142	M142		M142
	R143	R143		R143	R143							R143
				S146								S146
					H154							H154
	Q211		Q211			Q211		Q211		Q211		Q211
					L218							L218
	P240S†	P240S	P240S	P240S	P240S	P240S	P240S	P240S	P240S	P240S	P240S	P240S

*One herd represented by 30 animal samples could not be specifically classified as the information given by farmer was “Saanen, Toggenburg, Alpine, Anglo-Nubian”; six animals were described as ‘British goat’, one as ‘mix’ and two were Bagot

†The variation in codon 240 occurs in different combinations with the other polymorphisms and is also present in all breeds as a polymorphism of the wild-type allele: IRRQ-S_240_ or IRRQ-P_240_

There were 1293 collected samples with an average of 24.8 goat samples per herd (male to female ratio: 1:3). A total of 791 samples (61.2 per cent) were taken as blood by veterinary or scientific staff and 502 samples (38.8 per cent) were collected as nasal swaps by goat keepers. The final success rates for genotyping were 95.3 per cent and 97.4 per cent for blood and swap samples, respectively. However, the need to repeat the analysis for blood samples was higher than for swap samples.

Out of the 1293 samples, 1236 had a genotype result available (95.6 per cent of collected samples) of which 8 animals were found to be heterozygous QK222 goats (0.64 per cent genotype frequency, 95 per cent CI 0.28 to 1.27 per cent) while no homozygous KK222 goats were detected in the survey (Table 1). This gives a total K222 allele frequency of 0.32 per cent for this survey population. The eight QK222 goats were found in six herds (11.5 per cent of all sampled herds, 95 per cent CI 2.8 to 20.2 per cent). In one herd, two QK222 British Saanen goats were found, two QK222 Anglo-Nubian in another herd, and in four different herds, a single QK222 goat was found of three different breeds: British Saanen, British Toggenburg and crossed Saanen X British Toggenburg. All the herds were located in England: one in Berkshire, one in Yorkshire, one in North Yorkshire and two in Somerset. By breed this represents a 0.6 per cent allele frequency in Saanen, Toggenburg and their crossbreds, and 0.8 per cent for Anglo-Nubian goats. Seven of the eight QK222 goats were females, of which three were classified as breeding stock, while the other four were production (dairy) goats. The only QK222 billy goat was a British Saanen (Table 2). The 573 dairy goat samples came from 25.2 per cent males and 74.8 per cent females providing evidence against any sex bias for the K222 carriers.

Most British breeds encode on average four *PRNP* alleles, that is, wild-type sequences IRRQ-S240 or IRRQ-P240 and 2 of the 10 variant alleles that are known to exist in the British goat population. Some breeds, like the Boer goats, carry four variant alleles, whereas others such as Angora are less variable with only one variant allele.

For 748 DNA samples representing all breeds (equivalent to 1496 chromosomes), the ORF was sequenced in full so that potentially novel mutations could be identified and heterozygotes or homozygotes recorded. Novel polymorphisms were not found, but the polymorphism W102G has not been described in British breeds before. The allele frequencies are shown in Table 3 and compared with the previous survey (Goldmann and others 2011). The frequency of all variant alleles together was 37.3 per cent, which results in genotype frequencies as follows: of the 59.2 per cent that carried a variant of PrP, 45 per cent were single heterozygotes, 5 per cent were double heterozygotes and 10 per cent were homozygotes.

**TABLE 3:** *PRNP* allele frequencies (%) for two goat surveys in Great Britain

Collection period	2007–2009	2013–2014	
Animals	1195	748	
Chromosomes	2390	1496	
Polymorphism	[%]	[%]	Change
Wild-type	62.7	62.7	0
R101	0.1	0.1	0
G102	0	0.3	+0.3
S127	6.4	4.6	−1.8
M142	22.6	15.8	−6.8
R143	1	2.1	+1.1
S146	3.6	8.2	+4.6
H154	0.1	0.3	+0.2
Q211	2.3	5.1	+2.8
L218	0.4	0.3	−0.1
K222	0.9	0.5	−0.4

The authors genotyped codon 146 for 172 Boer goats across nine herds and found 103 carrying the S146 allele, 81.6 per cent were NS146 heterozygotes and 18.4 per cent SS146 homozygotes (Table 4); none carried the D146 allele. The allele frequency of 35.5 per cent in Boer goats is significantly different (P<0.0001) from that of the other breeds in this study, which was 1.3 per cent of 405 analysed genotypes. Genotypes for codons 142 and 211 were established for 742 and 784 goats, respectively. The M142 and Q211 alleles confer partial resistance and their frequency was therefore of interest. The M142 allele had high frequencies in Saanen (30.1 per cent, 12 herds) and Toggenburg (34.4 per cent, 7 herds) including the crossbred, with a substantial number of MM142 homozygotes (9 per cent and 13 per cent, respectively). All other goats together reached a 3 per cent allele frequency. The Q211 allele frequencies were particularly high in the Alpine and Golden Guernsey breeds with 15.6 per cent (three herds) and 68.1 per cent (three herds), respectively; all other breeds together had an allele frequency of only 0.9 per cent.

**TABLE 4:** *PRNP* genotype frequencies

Genotype	Goats (n)	%
GG127	640	90.6
GS127	61	8.6
SS127	6	0.8
II142	540	72.8
IM142	167	22.5
MM142	35	4.7
NN146	78	41.9
NS146	90	48.4
SS146	18	9.7
RR211	725	92.5
RQ211	41	5.2
QQ211	18	2.3
QQ222	1228	99.3
QK222	8	0.7
KK222	0	0

## Discussion

Breeding for scrapie resistance in goats is a long held aim for countries in which this prion disease has significant impact on their livestock production and animal welfare. The implementation of classical scrapie eradication programmes for sheep in European countries proved that it is possible to significantly reduce the prevalence of scrapie using *PRNP* genetic approaches in conjunction with herd cull protocols (EFSA 2014). This has been demonstrated by applying the appropriate dissemination of resistance alleles from the male breeding population, resulting in a large increase in the frequency of resistance alleles. For example, in Cyprus 95 per cent of the sheep population are now homozygous or heterozygous carriers of the resistant ARR allele, a massive increase from its 2005 level of 54 per cent (EU TSE Annual Report 2013).

In goats, there are two strongly protective *PRNP* alleles based on the K222 and S146/D146 polymorphisms. The authors’ survey has shown again that for the British goat population the S146 PrP allele is mostly limited to the Boer breed, where it is, however, found at a high frequency. In contrast, the K222 allele has been found in all major dairy breeds in the GB population, but at a very low frequency. Their analysis of 1236 goats from different regions of Britain for the K222 allele revealed only eight carriers in 52 herds, a very low allele frequency of 0.6 per cent and a herd frequency of 11.5 per cent. No KK222 homozygotes were found, which is in line with the low allele frequency. When compared with the British goat survey conducted in 2007–2009 (Goldmann and others 2011), Saanen goats and their crossbreds had a very similar allele frequency of 0.7 per cent, implying that without active *PRNP* genotyping and selection there may not be a significant increase in the K222 allele frequency.

Classical scrapie is commonly found in animals with homozygous IRRQ/IRRQ (wild-type) genotype. In goats, many *PRNP* alleles appear to have a protective effect mostly by extending incubation periods. Heterozygosity in the expressed PrP variants may be sufficient to lower the prevalence of disease by extending the incubation period, and they will, therefore, be of advantage for animals with a restricted lifespan in a commercial herd. With this in mind, it may not be surprising that of the 748 fully genotyped goats, 59.2 per cent were carriers of a variant *PRNP* allele.

Notable changes in the allele frequencies compared with the previous study were observed for M142, S146 and Q211. Although it is most likely that these overall differences between the surveys are associated with a different composition regarding the goat breeds, there may be real frequency movements in the breeds. For instance, Boer goats contributed almost twice as many (20.6 per cent) as before and they were the main carriers of the S146 allele leading to the increase to 8.2 per cent, at the same time they did show a low M142 allele frequency, which reduced the overall M142 allele frequency to 15.8 per cent. Similarly, the increase of the Q211 allele frequency appears to be due to the three times larger number of Golden Guernsey surveyed in this study (3 per cent) and the very high frequency (80 per cent) of this allele in this breed. Several of the other variant alleles appear to be breed specific, for example, L218 and H154 in Cashmere and G102 in Golden Guernsey. To accurately assess the allele frequency of these alleles, a larger number of herds and animals would have to be collected.

This survey has shown that the potentially scrapie-protective K222 allele is very rare in British goats and that there has not been a major change since the last survey six years ago when the breed composition of the goat sampling is taken into account. However, there were a larger proportion of herds containing at least one goat with the K222 allele. Breeding for K222-associated resistance to scrapie will be challenging and may demand a strict breeding programme using natural mating in those breeds where the K222 allele was found. Alternatively, the use of artificial insemination has to be contemplated with imported semen from K222 males of the targeted breeds. For those breeds where no K222 allele holders were found, private initiatives from breeding societies to genotype billy goats and/or importing semen or breeders may be considered.

The difficulty in recruiting the target number of herds has resulted in less precise estimates, adding a greater degree of uncertainty on the actual allele frequencies in the goat population, especially to the number of herds holding goats with potentially resistant alleles (up to 20 per cent). Goat keepers interested in producing breeding stock and interested in adding value to their goats may have been more likely to participate. On the other hand, farmers may have been less keen to participate with suspicion that the samples could be used to test for scrapie or reluctance to sample goats without a diagnostic purpose. However, given the fact that breeding for resistance for scrapie in goats is an unknown practice in Great Britain, it is unlikely that the selection of farmers may have introduced bias in the authors’ estimates.

Despite the low frequency of the potentially resistant K222 allele and the apparent low incidence of scrapie in British goats, the potential of breeding of resistance to scrapie in the goat population is an activity worth promoting among goat keepers, breeding societies and other stakeholders. It is important to note that the scrapie-infected herds detected in Great Britain have presented very high incidence of cases and suffered economic losses caused by decreased productivity, disruption of business operations due to official restrictions and compromised welfare. A genotype-based approach to eradicate scrapie is only an option for sheep according to current EU regulation (EC) No 999/2001 of the European Parliament and of the Council laying down the rules for the prevention, control and eradication of certain TSEs with its amendments. The fact that few goats with the K222 allele have been identified does not preclude the possibility to design and implement successful breeding programmes at national level in goats.
